# Pressure Sensitivity Enhancement of Porous Carbon Electrode and Its Application in Self-Powered Mechanical Sensors

**DOI:** 10.3390/mi10010058

**Published:** 2019-01-16

**Authors:** Keren Dai, Xiaofeng Wang, Zheng You, He Zhang

**Affiliations:** 1 ZNDY of Ministerial Key Laboratory, School of Mechanical Engineering, Nanjing University of Science and Technology, Nanjing 210094, China; dkr@njust.edu.cn; 2 Beijing Innovation Center for Future Chips, Department of Precision Instrument, Tsinghua University, Beijing 100084, China; yz-dpi@tsinghua.edu.cn

**Keywords:** porous electrode, pressure sensitivity, self-powered sensors, mechanical impact

## Abstract

Microsystems with limited power supplies, such as electronic skin and smart fuzes, have a strong demand for self-powered pressure and impact sensors. In recent years, new self-powered mechanical sensors based on the piezoresistive characteristics of porous electrodes have been rapidly developed, and have unique advantages compared to conventional piezoelectric sensors. In this paper, in order to optimize the mechanical sensitivity of porous electrodes, a material preparation process that can enhance the piezoresistive characteristics is proposed. A flexible porous electrode with superior piezoresistive characteristics and elasticity was prepared by modifying the microstructure of the porous electrode material and adding an elastic rubber component. Furthermore, based on the porous electrode, a self-powered pressure sensor and an impact sensor were fabricated. Through experimental results, the response signals of the sensors present a voltage peak under such mechanical effects and the sensitive signal has less clutter, making it easy to identify the features of the mechanical effects.

## 1. Introduction

In recent years, self-powered mechanical sensors have attracted wide attention for their applications in wearable smart devices and intelligent microsystems with limited energy supply [1,2,3,4,5]. For example, a self-powered pressure sensor can achieve skin-like tactile sensing without a power source, which benefits energy saving and promotes practical applications such as electronic skin [6,7,8]. A self-powered impact sensor can detect the characteristics of a target hit by an artillery shell, thus promoting the intelligence of weapon microsystems (such as a smart fuze) with limited volume and power supply [9].

Traditional self-powered mechanical sensors applied in measurements of static pressure and dynamic impact are mainly piezoelectric sensors [10,11,12], some of which benefit from porous materials [13,14]. However, due to the inherent physical properties of piezoelectric crystals, high frequency oscillations in the sensitive signals under mechanical effects are unavoidable, and complex signal processing must be applied to obtain the features of the target (e.g., the number of impacts during a continuous impact process) [15,16]. These insurmountable shortcomings make it urgent to develop mechanical sensors based on new principles.

In recent years, the dual functions of energy storage and piezoresistive sensitivity in porous electrodes, such as activated carbon and carbon nanotubes, have been investigated [17], and have become a new hotspot in research of self-powered mechanical sensors. For static pressure sensing, Zhang et al. realized a fusion of supercapacitors and self-powered pressure sensors using a carbon nanotube–polydimethylsiloxane film [18]. For dynamic impact sensing, the previous research of our team realized a novel device combing a supercapacitor and impact sensor based on an activated carbon film [19,20]. The sensitive signal has fewer high frequency oscillations, making it easier to identify target features. This unique characteristic is referred to as “self-filtering”, and a theoretical model was proposed to explain it in our former research [21].

For these self-powered mechanical sensors, the material properties of porous electrodes have a decisive influence on their energy storage density and mechanical sensitivity [18,20,21]. However, the methods for enhancing the mechanical sensitivity of porous electrodes have not been systematically studied, so that the optimal design and practical application of these self-powered mechanical sensors are limited for better performance.

In this paper, an enhancement method for pressure sensitivity of porous electrodes is proposed. An elastic polymer is added to the porous energy storage material, which forms a spatial network skeleton structure at the microscopic level by a shearing process. On the one hand, the micro-elastic skeleton structure can improve the elasticity of the electrode, so that the electrode can withstand greater stress; on the other hand, the deformation effect of the elastic polymer skeleton under mechanical effects results in a more significant change in the contact state between the conductive particles, and thus enhances the mechanical sensitivity of the electrode. Finally, based on the proposed high elastic piezoresistive film, a self-powered mechanical sensor was fabricated, and its superior sensing performance was experimentally verified. In general, the preparation process of the porous electrode proposed in this paper achieves effective enhancement of pressure sensitivity, and promotes practical applications of the self-powered impact sensor, based on the piezoresistive effect of the porous electrode.

## 2. Materials and Methods

**Figure 1:**
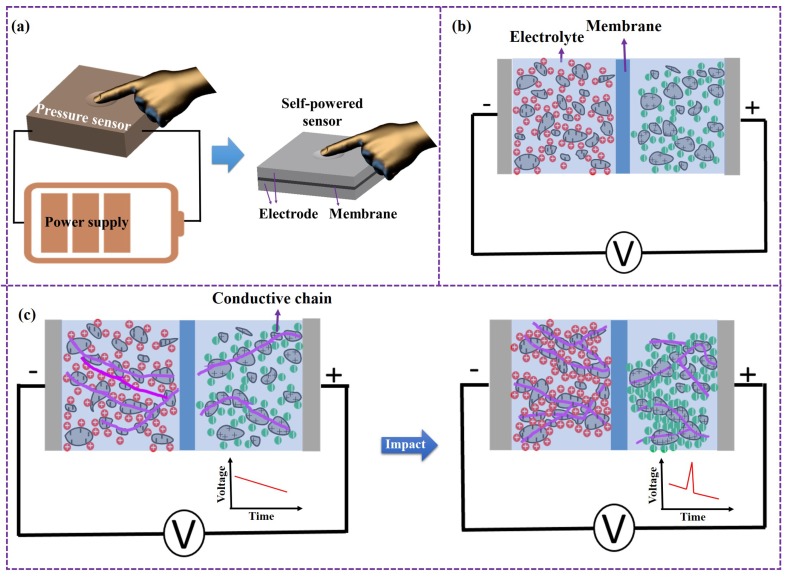
Diagram and mechanism of self-powered mechanical sensors. (**a**) A self-powered mechanical sensor works without a power supply. (**b**) Based on a porous electrode, energy storage is realized by the electric double-layer effect. (**c**) Based on a porous electrode, mechanical sensitivity is realized by the piezoresistive effect.

As shown in Figure 1a, traditional mechanical sensors need an external power source, which can be much larger than the sensor itself. In this paper, a self-powered mechanical sensor that is composed of two porous electrodes separated by a membrane is proposed, and it simultaneously achieves superior performance for both energy storage and mechanical sensitivity. This novel self-powered mechanical sensor has broad application prospects in the fields of electronic skin, intelligent fuzes, and so on. With the material modification in this paper, the flexibility of the sensor can be significantly enhanced. So, it can be used as a pressure sensor for electronic skin, realizing a tactile sensing for a robot. Furthermore, with a compact package, it can also serve as a high acceleration impact sensor to detect the structural characteristics of an attack target for an intelligent fuze, realizing intelligent ignition control of a projectile [22].

On the one hand, the porous electrode enables energy storage and provides energy supply during impact, as shown in Figure 1b. The porous electrode is composed of activated carbon and carbon nanotubes (or other porous materials); there are a lot of microscopic pores inside, which are filled with electrolyte (e.g., sulfuric acid). When the device is charged, an electrochemical double-layer structure forms at the microscopic solid–liquid interface due to the adsorption force between positive and negative ions, which realizes energy storage [23]. After being fully charged, the device can be discharged with certain current similarly to a power source.

On the other hand, the mechanical sensitivity of the device is achieved due to the piezoresistive effect of the porous electrode. As shown in Figure 1c, the conductivity of the porous electrode is determined by the number of micro-conductive chains. Under mechanical effects, the electrode will be deformed due to its loose microstructure [20,21], and the conductive particles are more likely to be closer to each other, increasing the number of conductive chains and greatly enhancing the conductivity of the electrode. The change in conductivity is reflected in the change of output voltage, thus realizing the perception of the mechanical effect.

Therefore, enhancing the piezoresistive performance of porous electrodes is the key to realizing self-powered mechanical sensors, and the enhancement can be achieved through the optimization of the micro-morphology of the porous electrode. Previous literature [20] has studied the relationship between electrode conductivity and porosity according to the general equivalent medium equation [24]:

(1) $$\sigma_{m}=\left\{\begin{array}{cc} {\left(\frac{\Phi -\Phi_{c}}{1-\Phi_{c}}\right)}^{t_{m}}\sigma_{h}, & \Phi_{c}\leq \Phi \leq 1\\ 0, & 0<\Phi <\Phi_{c} \end{array}\right.,$$

where $\sigma_{m}$ represents the conductivity of the porous electrode, $\sigma_{h}$ represents the conductivity of the carbon particles, Φ represents the volume ratio of the carbon particles, and $\Phi_{c}$ represents the critical volume ratio, $t_{m}$ is a coefficient.

**Figure 2:**
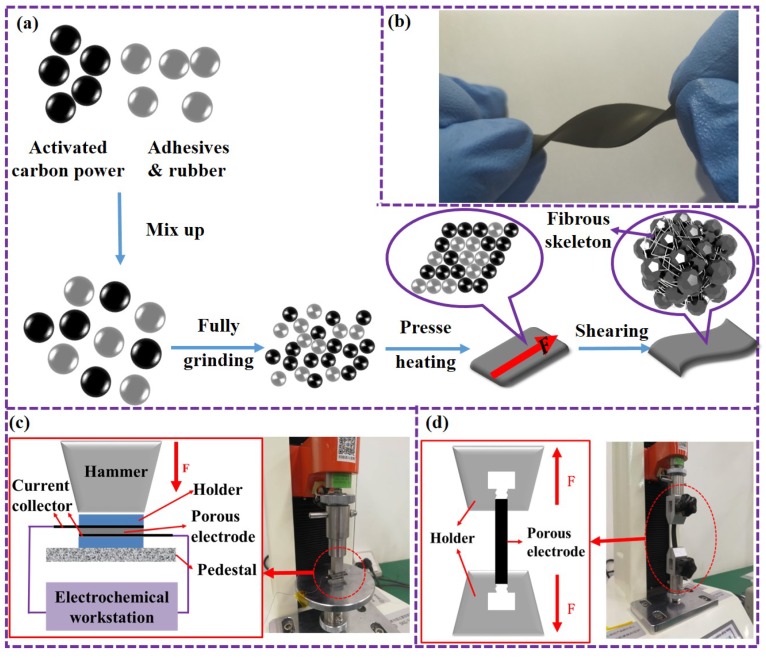
Preparation and testing of the porous electrodes. (**a**) Preparation process for microstructure formation. (**b**) Flexibility of the electrode under extreme torsion. (**c**) Experimental platform for piezoresistive testing. (**d**) Experimental platform for tensile testing.

According to Equation (1), a larger porosity is critical to improve the piezoresistive performance of the porous electrode, so an electrode with loose microstructure is preferred. However, for normal carbon electrodes, the large porosity also results in weak mechanical strength of the electrode, making it easy to be broken under mechanical effects. In order to solve this contradiction, a novel preparation process is proposed, forming an elastic micro-networked structure with polytetrafluoroethylene and achieving superior piezoresistive performance and elasticity simultaneously. The specific steps of the film preparation process are as follows (Figure 2a):

1. Mix activated carbon powder and a binder powder (e.g., polytetrafluoroethylene and rubber) evenly by concussion and grinding. The mass ratio of the activated carbon powder and the binder powder is 85:15.
2. The mixed powder is lightly pressed under high temperature heating (150–180 ${}^{\circ }C$) to form a film.
3. A shearing force is applied along the surface of the film, and the elastic binder and rubber component in the film are stretched. A micro-networked structure is formed, which has an envelope effect on the activated carbon micro-particles.

The porous electrode prepared by the above process has good elasticity, as shown in Figure 2b, and thus can be applied to flexible electronic devices such as electronic skin. In the following, in-depth tests and analyses of the piezoresistive characteristics of the prepared porous electrode are carried out. The experimental platform is composed of a mechanical press/tensile machine and an electrochemical workstation, as shown in Figure 2c,d. The experimental platform for the piezoresistive characteristics of the electrode film is shown in Figure 2c. Pressure is applied to the electrode film by a press machine, and the resistance change in the electrode is recorded by the electrochemical workstation. The experimental platform for the elasticity of the electrode film is shown in Figure 2d. The electrode film is stretched by a tensile machine until it breaks. The amount of tension and the length at which the film is stretched (i.e., the distance that the clamp moves) is recorded by the tensile machine.

## 3. Results

### 3.1. Pressure Sensitivity Enhancement of the Porous Electrode by Shearing

**Figure 3:**
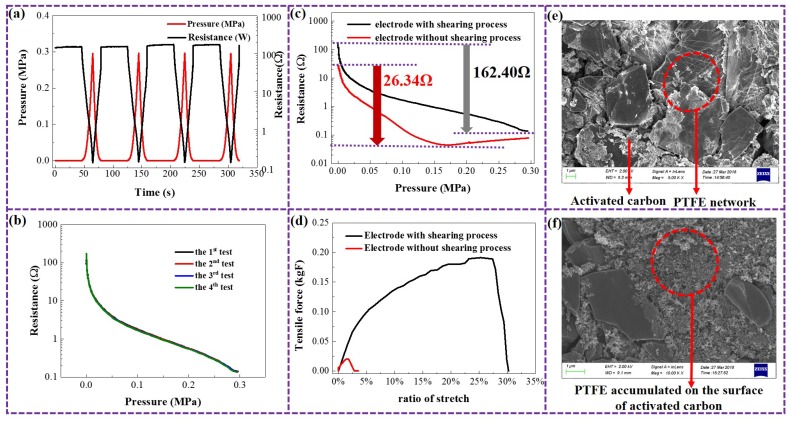
Changes in the microstructure and macroscopic properties of the porous electrodes by a shearing process. (**a**) Resistance change of the electrode during four consecutive pressurization– depressurization processes. (**b**) Repeatability of electrode piezoresistive characteristics during four consecutive pressurization–depressurization processes. (**c**) Piezoresistive characteristics of the electrodes with and without the shearing process. (**d**) Elasticity of the electrodes with and without the shearing process. (**e**) Electron micrograph of the electrodes with the shearing process where PTFE is the abbreviation of polytetrafluoroethylene. (**f**) Electron micrograph of the electrodes without the shearing process.

Four consecutive processes of pressurization and depressurization were applied to the electrode film. As shown in Figure 3a, when the pressure increases from 0 MPa to 0.3 MPa, the resistance of the electrode (10 mm × 10 mm × 1 mm) decreases from more than 100 Ω to less than 1 Ω. When the pressure is removed, the resistance almost returns to the original value. Figure 3b further illustrates that the resistance–pressure curves corresponding to the four consecutive pressurization–depressurization processes are almost coincident, verifying the reliability and stability of the electrode’s application for mechanical sensing based on its piezoresistive characteristic.

In the electrode preparation process in Figure 2a, the shearing process along the surface of the film is the key to modifying the material for the electrode. As shown in Figure 3c, the electrode with the shearing process has much better piezoresistive sensitivity and a wider sensitive range than the electrode without the shearing process. When the pressure is increased from 0 MPa to 0.3 MPa, the former’s resistance drops by 162.40 Ω, and its resistance decreases with the increasing pressure, until 0.3 MPa. However, the latter’s resistance changes by only 26.34 Ω, and stops decreasing around 0.15 MPa. In addition, the elasticity characteristics of the two are completely different. As shown in Figure 3d, the electrode film (50 mm × 10 mm × 1 mm) with the shearing process can withstand a tensile force up to nearly 0.2 kgF, and fracture occurs when the length of the stretch exceeds 25% of the original length. On the other hand, the electrode film without the shearing process can only withstand a tensile force of less than 0.02 kgF, and fracture occurs when the length of the stretch is only about 2% of the original length.

This tremendous improvement in piezoresistive sensitivity and elasticity is due to the changes in the microstructure of the porous electrode materials by the shearing process. As shown in Figure 3e, a loose micro-networked skeleton structure is formed in the electrode with the shearing process, which has an envelope effect on the particles distributed throughout the material. Energy spectrum tests show that the content of fluorine in the network skeleton structure is significantly higher than that in the granular particles, and it can be presumed that the network skeleton structure is mainly formed by the polytetrafluoroethylene binder under the shearing force. Since the polytetrafluoroethylene has good elasticity and its network skeleton structure has a loose microstructure, significant micro-deformation and resistance changes can be realized under pressure. At the same time, the superior elasticity property of the polytetrafluoroethylene network skeleton also enables the electrode to withstand larger stress and deformation. On the contrary, for the electrode without the shearing process, there are fewer microscopic network skeleton structures, but some cluster structures instead, as shown in Figure 3f. Energy spectrum tests show that the content of fluorine in the cluster structure is significantly higher than that of other regions, indicating that the binder has not been sufficiently dispersed during the preparation of the electrode film, but is clustered in some areas. As a result, the amount of binder in other areas was insufficient, making it easier to fracture under mechanical effects.

### 3.2. Pressure Sensitivity Enhancement of the Porous Electrode by the Rubber Component

**Figure 4:**
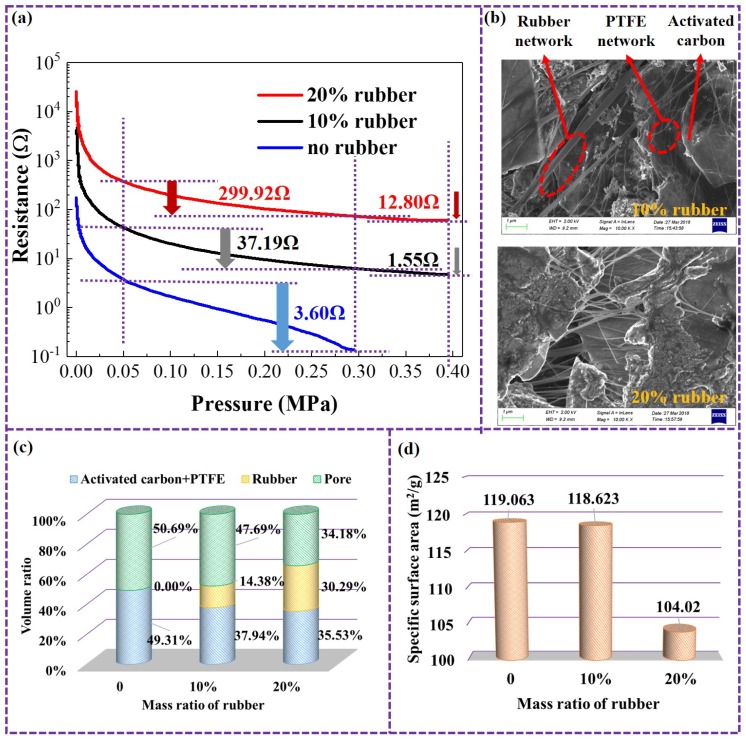
Changes in the microstructure and macroscopic properties of the porous electrodes by adding elastic rubber. (**a**) Piezoresistive characteristics of electrodes with different mass ratios of rubber. (**b**) Electron micrograph of the electrodes with different mass ratios of rubber. (**c**) Volume ratio of electrodes with different mass ratios of rubber. (**d**) Specific surface area of electrodes with different mass ratios of rubber.

Further experimental results reveal the more significant influence of rubber additives on the piezoresistive characteristics of the porous electrode. As shown in Figure 4a, as the mass fraction of Ethylene-Propylene-Diene Monomer (EPDM) rubber was increased from 0% to 20%, the magnitude of the resistance drop in the porous electrode, when the pressure increased from 0.05 MPa to 0.3 MPa, increases significantly. In addition, the sensitivity range of the electrode in response to the pressure is also broadened. For the electrode without rubber, the resistance remains at a small value without change when the pressure is increased to 0.3 MPa; while for the electrode with rubber mass fractions of 10% and 20%, when the pressure increases from 0.3 MPa to 0.4 MPa, the resistance continues to drop by 1.55 Ω and 12.8 Ω, respectively. This remarkable modulation of the piezoresistive effect also resulted from the changes in the microstructure of the porous electrode. As shown in Figure 4b, the electron micrograph of the porous electrode with rubber has two different network skeleton structures: one that is similar to the polytetrafluoroethylene skeleton in Figure 3e, and another network skeleton that is obviously more robust. Through energy spectrum tests, the fluorine content in the robust skeleton structure is significantly lower than the polytetrafluoroethylene skeleton structure in Figure 3e. So, it can be presumed that these robust network skeletons are formed by the rubber stretched under the effect of the shearing force. Since the elasticity of the network skeleton structure formed by rubber is much stronger than the polytetrafluoroethylene network skeleton, it has a more significant influence on the piezoresistive characteristics of the porous electrode.

Although the increase in the rubber component can improve the piezoresistive characteristics of the porous electrode, it also causes a loss of its energy storage characteristics. As shown in Figure 4c, as the mass ratio of rubber increases, the volume ratio of the microscopic pores of the electrode decreases remarkably, resulting in a loss of specific surface area, as shown in Figure 4d. The loss of specific surface area means that the micro-interface of the electrochemical double-layer energy storage structure is reduced [25]:

(2) $$i_{\mathrm{DL}}=a_{v}C_{\mathrm{dl}}\frac{\partial \left(\Psi_{s}-\Psi_{l}\right)}{\partial t},$$

where $i_{\mathrm{DL}}$ represents the current density resulting from electric double-layer effect, $a_{v}$ represents the specific surface area of the electrodes, and $C_{\mathrm{dl}}$ represents the capacitance associated with the electric double-layer effect. $\Psi_{s}$ and $\Psi_{l}$ represents the potential of the electrode phase and electrolyte phase, respectively.

So, as the mass ratio of the rubber increases, the energy storage performance of the porous electrode decreases, which is not conducive to self-powered mechanical sensors. Therefore, a moderate amount of rubber component is preferred to keep a balance between the energy storage characteristics and the piezoresistive characteristics. In the following section, with a prototype assembly and performance test, a porous electrode with a rubber mass ratio of 10% is chosen.

### 3.3. Performance of Applications in Self-Powered Mechanical Sensors

With flexible and compact packages, a self-powered pressure sensor and a self-powered impact sensor, based on the porous electrodes with piezoresistive sensitivity enhancement, are both realized.

**Figure 5:**
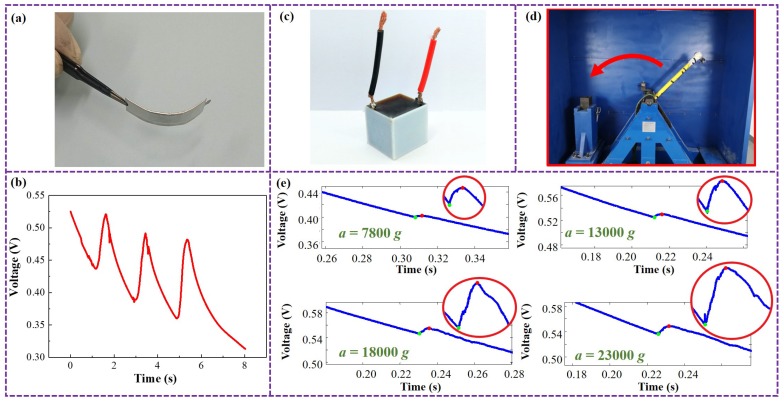
Applications as self-powered mechanical sensors. (**a**) The flexible pressure sensor device. (**b**) Response signal of the pressure sensor under continuous pressing. (**c**) The impact sensor device. (**d**) The Machete Hammer system. (**e**) Response signal of the impact sensor under different accelerations of impact.

A picture of the self-powered pressure sensor is shown in Figure 5a. With superior flexibility, this sensor can be applied to flexible electronic devices such as electronic skin. Figure 5b shows the sensitive signal of the sensor under continuous pressing. It is obvious that the voltage of the sensor increases and forms a peak with almost no cluttering data during each pressing process, and the continuous pressing process can be clearly identified. The amplitude of the sensitive voltage peak signal reaches about 10 mV, which is enough for signal acquisition and processing, verifying that the piezoresistive enhancement proposed in this paper is effective for applications.

A picture of the self-powered impact sensor is shown in Figure 5c. Its impact sensitivity characteristics are tested by a Machete Hammer system which utilizes the potential energy of the counterweight to produce an ultra-high impact of up to 30,000 g, as shown in Figure 5d. The experimental results are shown in Figure 5e, The response signal of the sensor has a clear voltage peak when it is subjected to a high-*g* impact, with a wide range from 7800 g to 23,000 g. Considering that the impact process in practical applications is about 1 millisecond [26], the sampling rate for signal acquisition of the sensor is set as 50 kHz. The voltage peak has almost no clutter even at such a high sample rate, showing superior impact feature recognition ability over traditional piezoelectric sensors.

The fewer high frequency components in the response signal of the self-powered sensor is due to its essential ’self-filtering’ mechanism, which has been revealed in a previous study [21]. The response of the porous electrode can be approximated by the vibration of the electrode in the electrolyte under an external force:

(3) $$f\left(t\right)-kx\left(t\right)-\mu \frac{dx(t)}{dt}=m\frac{d^{2}x\left(t\right)}{dt^{2}},$$

where f(t) is the external force as a function of time, *k* is the equivalent stiffness coefficient of the electrode, x(t) is the displacement as a function of time, μ is the equivalent damping coefficient of the electrolyte, and *m* represents the equivalent mass of the electrode.

To investigate the frequency components of x(t), a Fourier transformation is applied to both sides of Equation (3):

(4) $$F\left(\omega \right)-kX\left(\omega \right)-\mu j\omega X\left(\omega \right)=-m\omega^{2}X\left(\omega \right),$$

where F(ω) and X(ω) are the Fourier transformations of f(t) and x(t), respectively. Thus, the spectrum of displacement is:

(5) $$X\left(\omega \right)=\frac{F(\omega )}{k-m\omega^{2}+j\mu \omega }=\frac{\left|F(\omega )\right|}{\sqrt{{\left(m\omega^{2}-k\right)}^{2}+{\left(\mu \omega \right)}^{2}}}e^{j\Phi (\omega )},$$

where Φ(ω) represents the phase-frequency characteristics of X(ω).

The porous carbon electrode has a small equivalent stiffness coefficient and a large viscous damping coefficient (compared with a silicon beam structure and air damping in traditional impact sensors), which can effectively suppress high frequency components in the response signal according to Equation (5).

## 4. Conclusions

This paper presents a preparation process that can enhance the piezoresistive characteristics of porous electrodes. The experimental results show that by applying a shearing force along the surface of the electrode film, the binder is stretched to form a microscopic network skeleton structure. This has an envelop effect on porous particles such as activated carbon, and significantly enhances the piezoresistive characteristics and elasticity of the electrode. In addition, by adding a highly elastic rubber component, another more robust microscopic network skeleton structure is formed by the rubber under the shearing process, and a flexible porous electrode with superior piezoresistive characteristics and elasticity can be more effectively obtained. Based on such porous electrodes, both a self-powered flexible pressure sensor and a self-powered impact sensor were realized, with response signals of voltage peaks under pressure and high acceleration impact stimuli. Moreover, the sensors have superior ability to recognize features of mechanical processes, such as the number of consecutive pressing processes. These superior self-powered sensing characteristics provide new technological approaches for microsystems with limited power sources, such as electronic skin and smart fuzes.
